# Effect of a 2.45-GHz radiofrequency electromagnetic field on neutrophil chemotaxis and phagocytosis in differentiated human HL-60 cells

**DOI:** 10.1093/jrr/rru075

**Published:** 2014-09-05

**Authors:** Shin Koyama, Eijiro Narita, Yoshihisa Suzuki, Masao Taki, Naoki Shinohara, Junji Miyakoshi

**Affiliations:** 1 Laboratory of Applied Radio Engineering for Humanosphere, Research Institute for Sustainable Humanosphere, Kyoto University, Gokasho, Uji, Kyoto 611-0011, Japan; 2 Department of Electrical Engineering, Graduate School of Engineering, Tokyo Metropolitan University, 1-1 Minami Ohsawa, Hachioji, Tokyo 192-0397, Japan

**Keywords:** radiofrequency (RF), immune response, neutrophil, chemotaxis, phagocytosis

## Abstract

The potential public health risks of radiofrequency (RF) fields have been discussed at length, especially with the use of mobile phones spreading extensively throughout the world. In order to investigate the properties of RF fields, we examined the effect of 2.45-GHz RF fields at the specific absorption rate (SAR) of 2 and 10 W/kg for 4 and 24 h on neutrophil chemotaxis and phagocytosis in differentiated human HL-60 cells. Neutrophil chemotaxis was not affected by RF-field exposure, and subsequent phagocytosis was not affected either compared with that under sham exposure conditions. These studies demonstrated an initial immune response in the human body exposed to 2.45-GHz RF fields at the SAR of 2 W/kg, which is the maximum value recommended by the International Commission for Non-Ionizing Radiation Protection (ICNIRP) guidelines. The results of our experiments for RF-field exposure at an SAR under 10 W/kg showed very little or no effects on either chemotaxis or phagocytosis in neutrophil-like human HL-60 cells.

## INTRODUCTION

Mobile phone use has spread throughout the world. Wireless communication devices emit non-ionizing electromagnetic radiofrequency (RF) radiation in the frequency range of 300 MHz to 300 GHz. This has raised public concern regarding the increasing use of mobile phones and their potential health risks. Several epidemiological studies have suggested the possible risks of RF-field exposure [1–3]. However, few studies have shown adverse effects from RF fields [4, 5], and most cellular research indicates that non-thermal RF exposure does not cause an adverse biological effect [6–8]. In this study, an adverse effect indicates decreased immunological function, such as chemotaxis or phagocytosis, compared with normal healthy cells. For the classification of carcinogenesis, the International Agency for Research on Cancer (IARC) has categorized as follows: 1 (carcinogenic), 2A (probably carcinogenic), 2B (possibly carcinogenic), 3 (not classifiable as to it carcinogenic) and 4 (probably not carcinogenic). Presently, the IARC has classified RF fields as in group 2B [9]. No obvious adverse effects due to RF-field exposure have been obtained to date, although there is positive data for an effect on the immune system, which must be taken into consideration. Some articles indicate that cellular mobility is changed by exposure to RF fields [10, 11]. To resolve these discrepancies, we investigated the effects on the immune system in the form of changes in neutrophils exposed to RF fields.

The human immune system excludes exogenous materials to maintain homeostasis when foreign microbes invade. Neutrophils are the vital gatekeepers of a host's microbiome, defending against invading microbes. In this study, we investigated the effects of exposure to RF fields on chemotaxis and phagocytosis in human neutrophils differentiated from HL-60 cells.

## MATERIALS AND METHODS

### Cell culture

**Fig. 1.**
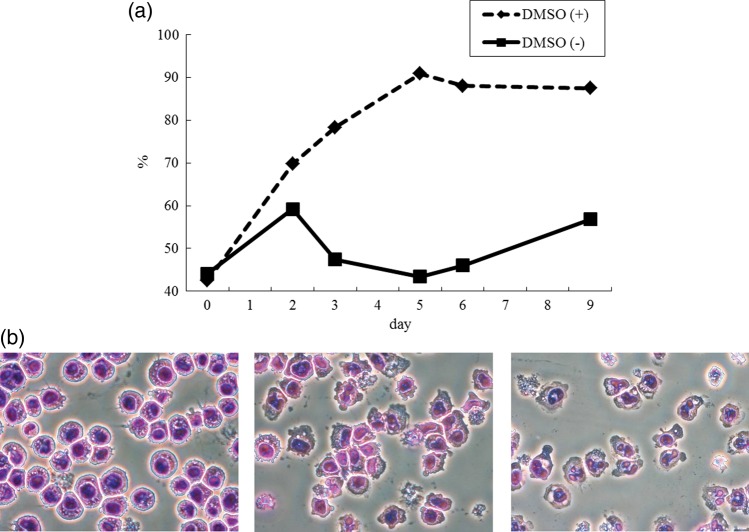
(**a**) Expression of CD11b in HL-60 cells after the treatment, with or without DMSO. (**b**) Morphological changes in HL-60 cells after treatment with DMSO. The segmented neutrophil-like cells were observed 3 and 6 days after the treatment.

Human leukemia HL-60 cells from a 36-year-old female (JCRB0085) were purchased by Japan Health Sciences Foundation (Tokyo, Japan) and were cultured in Roswell Park Memorial Institute (RPMI) 1640 medium supplemented with 10% inactivated fetal calf serum at 37°C in an atmosphere of 95% air and 5% CO_2_. HL-60 provides a unique *in vitro* model system for studying cellular events [12–14]. For differentiation, 1.25% dimethyl sulfoxide (DMSO) was added to 5 × 10^6^ cells in 38.1 ml of culture medium, followed by incubation for 3–9 days. Figure 1 shows the expression of CD11b as one of the differentiation markers, and morphological change, respectively, of HL-60 cells after treatment with DMSO. The cells differentiated to neutrophil-like cells after treatment with DMSO for 3 days.

### RF-field exposure

After the cells differentiated for 3 days, they were exposed to RF fields of 2.45 GHz at 2 and 10 W/kg specific absorption rates (SARs) for 4 and 24 h in a specially designed exposure apparatus (based on a cylindrical waveguide using TM01 mode, as previously reported) [15, 16]. Briefly, the apparatus consists of a cylindrical waveguide, the end of which is terminated by a short-circuiting metallic plate (which generates standing waves) and a signal generator (E4438C ESG, Agilent Technologies, Santa Clara, CA, USA). The dosimetry of the RF fields was performed with both numerical and experimental approaches [15, 16]. A culture dish with an inside diameter of 90 mm was placed on the short-circuiting metallic plate inside the waveguide, where the atmospheric conditions were controlled appropriately for cell culture by introducing 5% CO_2_ and 95% humidified air. The temperature of the culture medium was controlled by a Peltier controller (TDC-1550; Cell System Co. Ltd, Kanagawa, Japan) and was maintained at 36.8 ± 0.4°C at the bottom of a culture dish.

### Migration potency

To measure the migration potency, neutrophil chemotaxis was performed using an EZ-TAXIScan chemotaxis apparatus (ECI, Inc., Tokyo, Japan) [17]. One microliter of the cells at a concentration of 2 × 10^6^/ml was applied to one of two compartments. Two concentrations (10^−7^ and 10^−8^M) of formyl-methionyl-leucyl-phenylalanine (fMLP) were applied to another compartment as a chemoattractant. Time-lapse images were recorded every 30 s for 30 min, and cell migration was analyzed using a TAXIScan Analyzer2 (ECI, Inc., Tokyo, Japan). In each experiment, 20 cells were assessed for cell migration speed and directionality, and then we calculated the average based on the movement of the 20 cells. The speed of cell migration was expressed in μm/s. The directionality of migration was expressed as the angle (in radians) toward the chemoattractant from the start line (i.e. π/2 indicates cells migrate toward the chemoattractant directly, and the value can be translated into 1.57).

### Phagocytosis

In this study, a flow cytometric technique was used to detect phagocytosis. This technique is described in previously published articles [18–20]. Aliquots of 100 μl of the cells at a concentration of 1 × 10^4^/μl were incubated with 10 μl of FluoSpheres Fluorescent Microsphere (1 × 10^10^ microspheres/ml Invitrogen F13081) for 40 min at 37°C. (In this procedure, neutrophils take in the microsphere particles through phagocytosis.) Next the cells were washed five times with PBS (to deplete the free particles) and then suspended with 1 ml of PBS. The cell suspensions were analyzed using a flow cytometer (Becton Dickinson, NJ, USA).

### Statistical analysis

Data were expressed as the mean ± S.D. Each experiment was performed three times, and statistical analysis was evaluated using the Mann–Whitney *U* test to compare the sham and RF-field exposure. *P* < 0.05 was considered to be statistically significant.

## RESULTS

### Effects of RF-field exposure on neutrophil chemotaxis

We investigated the neutrophils' chemotaxis after exposure to RF fields using the EZ-TAXIScan chemotaxis assay system, which is a widely used technique [21–23].

**Fig. 2.**
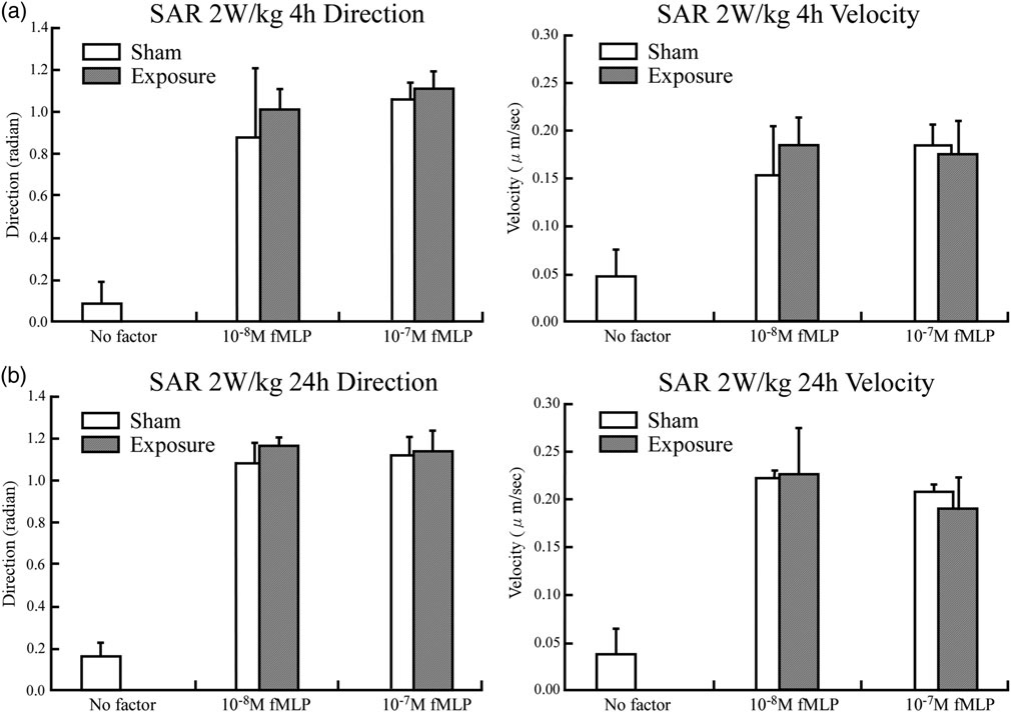
Chemotaxis in differentiated HL-60 cells were assayed using 0, 10 and 100 nM fMLP as the chemoattractant after exposure to RF fields at the SAR of 2 W/kg for (**a**) 4 h; (**b**) 24 h.

Figure 2 indicates the results for the neutrophils' chemotaxis in terms of migration speed and directionality. When fMLP (which stimulates the chemotaxis) was added, there was a statistically significant increase in the migration speed and directionality compared with no fMLP. However, the enhancement of the migration speed and the directionality was not detected in either the RF or sham exposures with fMLP treatment. The exposure to 2.45-GHz RF fields at 2 W/kg for 4 or 24 h did not affect the migration speed or directionality of neutrophils.

**Fig. 3.**
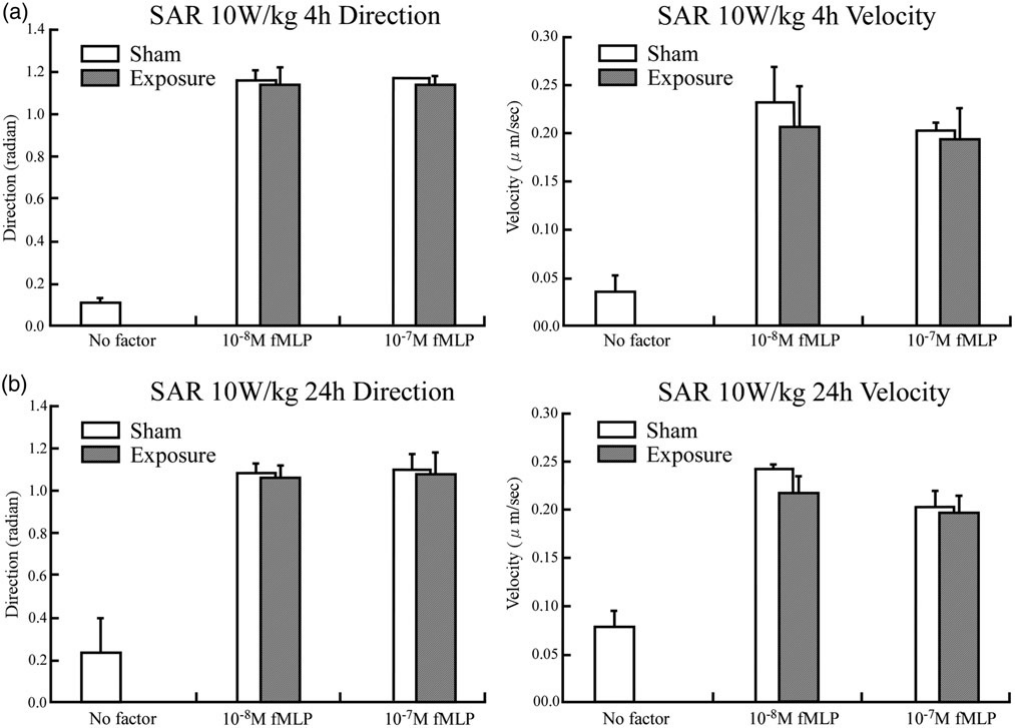
The differentiated HL-60 cells chemotaxis was assayed using 0, 10 and 100 nM fMLP as the chemoattractant after exposure to RF fields at the SAR of 10 W/kg for (**a**) 4 h; (**b**) 24 h.

The results for chemotaxis after exposure to RF fields at 10 W/kg for 4 and 24 h are shown in Fig. 3. Exposure to RF fields at 10 W/kg of SAR again did not produce any differences in either the migration speed or the directionality of the neutrophils.

### Effect of RF-field exposure on phagocytosis

**Fig. 4.**
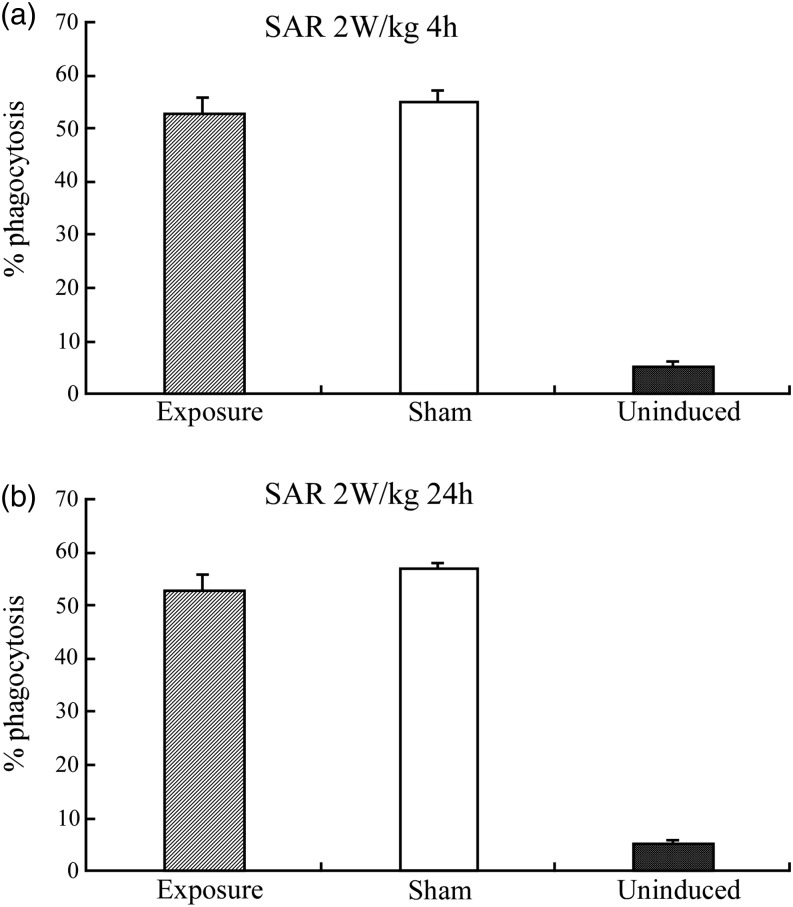
The percentage of phagocytosis in differentiated HL-60 cells after sham exposure or exposure to RF fields at the SAR of 2 W/kg for (**a**) 4 h; (**b**) 24 h.

Figure 4 indicates the percentage of phagocytosis using a flow cytometry technique to detect the fluorescent microparticles.

**Fig. 5.**
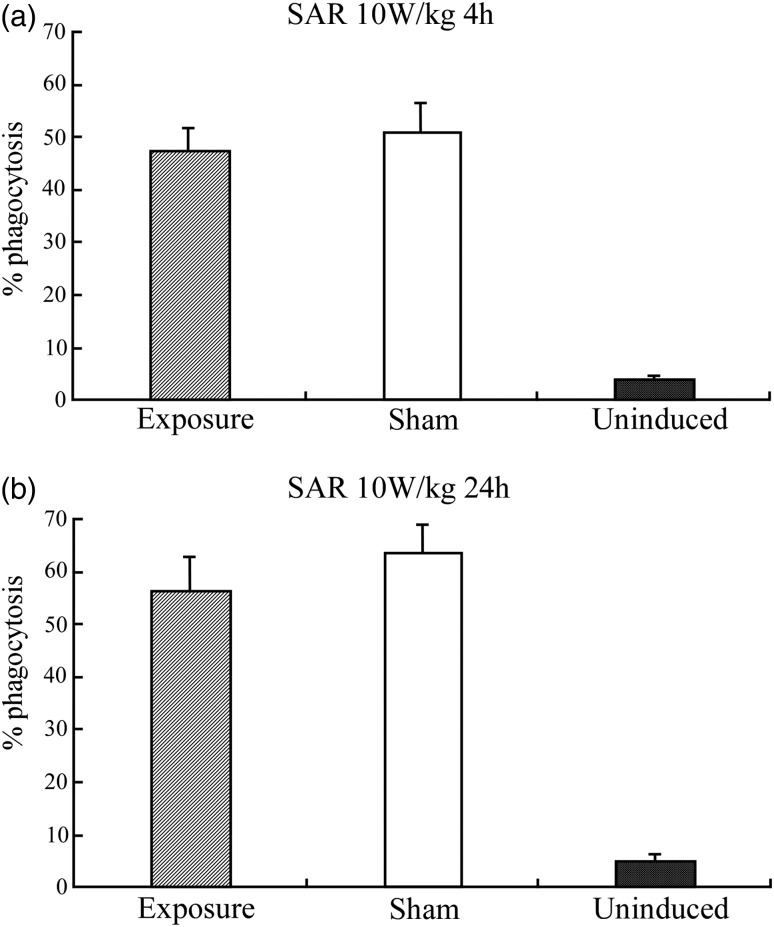
The percentage of phagocytosis in differentiated HL-60 cells after sham exposure or exposure to RF fields at the SAR of 10 W/kg for (**a**) 4 h; (**b**) 24 h.

Figure 5 shows the results for phagocytosis in cells exposed to RF fields at 2 W/kg for 4 and 24 h. The percentage of phagocytosis after exposure to RF fields was not significantly different from the percentage after sham exposure. In addition, exposure to RF fields at 10 W/kg did not affect the percentage of phagocytosis compared with the result after sham exposure.

## DISCUSSION

In this study, we evaluated the effects of RF-field exposure on chemotaxis and phagocytosis in human HL-60 cells using EZ-TAXIScan chemotaxis apparatus and FluoSpheres Fluorescent Microsphere, respectively. We could not detect any adverse effects on neutrophil migration or phagocytosis by RF-field exposure under any of the conditions investigated in this study.

Lai *et al*. previously reported that acute low-intensity microwave exposure increased DNA single-strand breaks [24]. Several studies have also shown that DNA strand breaks are increased by RF exposure [25–27]. However, many studies concluded that RF exposure does not cause DNA strand breaks [28–35]; thus, there is controversy over the cellular effect of RF exposure. There are numerous reports concerning genotoxic effects in cellular RF studies, but very few involving the cellular immune system for which there are positive data that indicate a significant change in the immune cells [10, 11]. This is why we investigated the potential role of RF-field exposure on the immune system. Neutrophils are the first to respond to foreign invaders by chemotaxis and phagocytosis. We measured the migration speed and directionality to evaluate the chemotaxis. Four types of RF-field exposures, namely at 2 and 10 W/kg SAR for 4 h (short term) and 24 h (long term) were used. We investigated a frequency of 2.45 GHz, which is widely used, and SAR of 2 and 10 W/kg, which are the limits set by Ministry of Internal Affairs and Communications for the general environment and the management environment in Japan, respectively. For none of the exposure conditions there was any significant difference between the sham exposure and RF-field exposure. This indicates that the RF-field exposure tested might have no adverse effect on neutrophil chemotaxis. However, Aly *et al.* indicated that after exposure to 900-MHz RF fields, neutrophil speeds increased by ∼50% compared with neutrophils at the same temperature without the RF [10]. These results were very different from our results, and may be either due to the cell preparation or to the difference in frequency. In our study, we used a differentiated HL-60 cell line, however Aly *et al.* used fresh human neutrophils. Another article by Tiwari and Singh indicated that 1800-MHz RF radiation from mobile phones affects the activity and behavior of human leukocytes [11]. They found a significant change in leukocyte behavior after exposure to RF fields. This effect may also depend on the difference in frequency or exposure time. In any case, we could not detect clear effects of exposure to RF fields. More detailed study is necessary to investigate the effect of RF fields on cell behavior.

We also investigated neutrophil phagocytosis—another aspect of our body's first immune response. We could not detect any difference in phagocytosis in cells exposed to sham exposure compared with those exposed to the RF fields.

The immune system protects hosts from infection and cancer. When an external organism invades the body, the immune cells start to attack the organism for self-protection. These cells produce many antibodies that inhibit the external threat, and killer T cells then eliminate the invader. Immune cells have an important role in this process, and the effect of RF fields on these cells has been examined. To date, there are few studies on the effects of RF fields and the human immune system. Tuschl *et al*. has reported that they found no statistically significant effects of exposure and that there is no indication that emissions from mobile phones are associated with adverse effects on the human immune system (IL-1, -2, and -4; INF-γ; and INF-α) [36].

Thorlin *et al*. examined the effect of a 900-MHz RF field on cultured astroglial and microglial brain cells [37]. Primary cultures enriched with astroglial cells were exposed to a 900-MHz RF field in a temperature-controlled exposure system at SARs of 3 W/kg (global system for mobile communication-modulated wave) for 4, 8 and 24 h or 27 W/kg continuous wave for 24 h, and the release into the extracellular medium of two proinflammatory cytokines (IL-6 and TNF-α) was analyzed. This study provided no evidence for any effect of the RF fields on damage-related factors in glial cells in culture.

A group in Poland reported positive effects of an RF field on immune cell activity. In an earlier study, G0-phase peripheral blood mononuclear cells (PBMCs) that were exposed to 1.3-GHz RF fields and subsequently cultured showed changed immune activity [38]. Stankiewicz *et al*. demonstrated that the microcultures of PBMCs exposed to an RF field (900 MHz, GMS, 27 V/m, SAR = 0.024 W/kg) had a significantly higher response to a mitogen and higher immunogenic activity of monocytes (LM index) than control cultures [39].

In our study, we demonstrated that neutrophil chemotaxis and phagocytosis is unaffected by RF fields at a relatively high intensity SAR. Since there is not enough research to definitively demonstrate the effect of RF fields on the immune system, further studies are required.

In recent times, the relationship between RF exposure and oxidative stress has been discussed [40–42]. Usselman *et al.* have reported an increase in free radical concentration in the presence of a 7-MHz RF magnetic field [43]. However, Lantow *et al*. examined the effect of an RF field on reactive oxygen species (ROS) production [44], and they detected no significant differences in free radical production after exposure to the RF fields (1800MHz, GMS, SAR = 0.5–2.0 W/kg) compared with the respective controls, and no additional effects on superoxide radical anion production were detected after co-exposure to RF fields together with TPA or LPS. The work by Zeni *et al*. concurs that there is no induction by 900-MHz RF-field exposure, either alone or in combination with 3-chloro-4-(dichloromethyl)-5-hydroxy-2(5H)-furanone, or induced formation of ROS under any of the experimental conditions investigated [45].

Although we demonstrated that RF exposure did not affect chemotaxis and phagocytosis on differentiated HL-60 cells in this study, further investigation would be needed to elucidate the relationship between RF exposure and human health. We should investigate Th1/Th2 balance or T-cell-dependent antibody response (TDAR) in order to evaluate another immunological response under the same conditions. These results indicate there is no adverse effect on the first immune response when the human body is exposed to 2.45-GHz RF fields at the SAR of 2 W/kg, which is the maximum value recommended by the International Commission for Non-Ionizing Radiation Protection (ICNIRP) guidelines [46].

## FUNDING

Funding to pay the Open Access publication charges for this article was partly provided by two grants a Grant from the Ministry of Internal Affairs and Communications, Japan, and Frontier Researches in Sustainable Humanosphere.
